# A Multi-Stage Model for Fundamental Functional Properties in Primary Visual Cortex

**DOI:** 10.1371/journal.pone.0034466

**Published:** 2012-04-09

**Authors:** Nastaran Hesam Shariati, Alan W. Freeman

**Affiliations:** Discipline of Biomedical Science, University of Sydney, Lidcombe, New South Wales, Australia; The University of Plymouth, United Kingdom

## Abstract

Many neurons in mammalian primary visual cortex have properties such as sharp tuning for contour orientation, strong selectivity for motion direction, and insensitivity to stimulus polarity, that are not shared with their sub-cortical counterparts. Successful models have been developed for a number of these properties but in one case, direction selectivity, there is no consensus about underlying mechanisms. We here define a model that accounts for many of the empirical observations concerning direction selectivity. The model describes a single column of cat primary visual cortex and comprises a series of processing stages. Each neuron in the first cortical stage receives input from a small number of on-centre and off-centre relay cells in the lateral geniculate nucleus. Consistent with recent physiological evidence, the off-centre inputs to cortex precede the on-centre inputs by a small (∼4 ms) interval, and it is this difference that confers direction selectivity on model neurons. We show that the resulting model successfully matches the following empirical data: the proportion of cells that are direction selective; tilted spatiotemporal receptive fields; phase advance in the response to a stationary contrast-reversing grating stepped across the receptive field. The model also accounts for several other fundamental properties. Receptive fields have elongated subregions, orientation selectivity is strong, and the distribution of orientation tuning bandwidth across neurons is similar to that seen in the laboratory. Finally, neurons in the first stage have properties corresponding to simple cells, and more complex-like cells emerge in later stages. The results therefore show that a simple feed-forward model can account for a number of the fundamental properties of primary visual cortex.

## Introduction

Fifty years of research have provided a detailed description of signal processing in mammalian primary visual cortex. We know, for example, that individual neurons are strongly selective for contour orientation, the spatial frequency of grating stimuli, and the direction of stimulus motion. Further, there is a subset of neurons – known as simple cells – that are sensitive to stimulus polarity and others – complex cells – that are relatively insensitive to polarity. The literature also describes the diversity of these properties across neuronal populations. Some cells, for example, are completely selective for the direction of stimulus motion, whereas other cells are indifferent to motion direction. The diversity of properties has been well documented for orientation selectivity [1], spatial frequency selectivity [2], direction selectivity [3], and for the simple cell/complex cell dichotomy [4].

The modelling of these properties has progressed in tandem with the accumulation of physiological results. There are models that successfully account for orientation selectivity and the existence of complex cells [5]–[10]. There is no agreement, however, about the physiological mechanisms underlying direction selectivity. It has long been recognised that at least two sensors are required and that these sensors must differ in their spatial locations and temporal signal-processing properties. Further, when the input is cyclic, there are advantages in having sensors that differ by a quarter of a cycle in both space and time [11], [12]. Saul and Humphrey [13] tested the temporal properties of relay cells in the lateral geniculate nucleus and showed that the response of lagged cells was delayed relative to non-lagged cells by approximately a quarter-cycle at low temporal frequencies. They therefore suggested that lagged and non-lagged cells could together provide the necessary inputs for cortical direction selectivity.

This *quadrature* hypothesis was thrown into doubt by Peterson et al. [3]. They recorded from direction-selective cells and modelled their responses by assuming that each cell sums two inputs that were not direction-selective. They found the latency difference of the inputs to be almost uniformly distributed between 0° and 90°, implying that lagged geniculate cells are not necessary for the generation of direction selectivity. There are also models for direction selectivity that include a contribution from intracortical circuitry (for example Ursino et al. [14]). Given that the sub-cortical timing is contentious, however, cortical involvement in generating direction selectivity becomes hard to interpret.

In this paper we describe a new model for direction selectivity. We take our lead from recent physiological evidence that the geniculate inputs to a column in the cat's primary visual cortex comprise a population of on-centre cells interspersed with a population of off-centre cells [15] and that the off-centre cells lead their on-centre counterparts by 3–6 ms [16]. Correspondingly, our model assumes that each cell in the first cortical stage receives mixed on- and off-centre inputs, with the latter leading by a few milliseconds.

More generally, we note that there are substantial shortfalls in previous modelling of other cortical properties, such as orientation selectivity and the emergence of complex cells. First, the models tend to focus on explaining a single functional property; each model therefore accounts for only a small subset of neural behaviour. Second, there have been few attempts to model the diversity of properties across neuronal populations. One notable exception is Ringach's model [17] for the variability of orientation selectivity.

We address both of these deficiencies in previous modelling work. We have two aims. The first is to describe the simplest possible model that can reproduce the orientation selectivity, spatial frequency selectivity, direction selectivity, and insensitivity to stimulus polarity, of neurons in primary visual cortex. The second aim is to find the extent to which the model can reproduce the diversity of these properties across a population of neurons. The simplicity of the model is illustrated by its starting point, which uses just two relay neurons in the lateral geniculate nucleus. Further, there are no feedback pathways in the model. This allows us to test how far a purely feed-forward model can be pushed to predict cortical properties.

To make the task manageable, the scope of the model is limited in three ways. First, given that the literature describing primary visual cortical function is richer for the domestic cat than for other species, we have chosen to model the cat's visual pathway. Second, there are several parallel sub-cortical pathways in the cat's visual system [18]; the model is restricted to the pathway with the highest spatial resolution, the X-cell pathway. Third, whereas primary visual cortex extends over more than one area in the cat, only area 17 is considered here because that is the major target for the X-cell pathway.

### Model design

We here describe the design of the model in broad terms. Model equations and parameters are provided in the Methods section. Two guiding principles were used in designing the model: simplicity, and adherence to known anatomy and physiology. The simplicity principle is illustrated by the starting point, which we call the basic model. This has just two sub-cortical pathways, one on-centre and the other off-centre. There is no surround mechanism, a lone cortical column, and sub-cortical signal processing is linear. The basic model is sufficient to produce the elements of the four cardinal functional properties described above. We then modify the model to improve its match with specific laboratory data. In accordance with the second principle, the model's parameters – such as ganglion cell concentration, and the centre mechanism size of geniculate receptive fields – are taken from published data.

### Model structure

**Figure 1:**
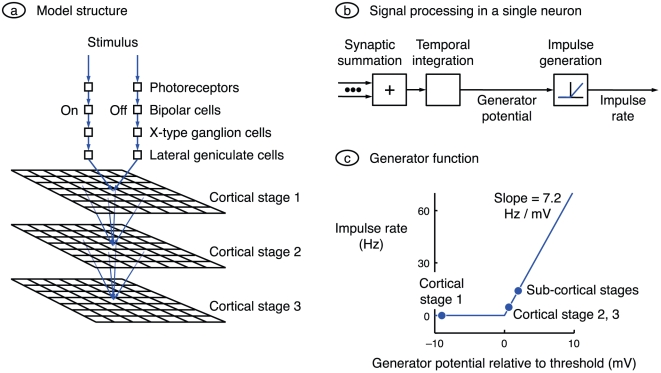
Model design. **a.** The basic model comprises two sub-cortical channels and three cortical processing stages. Each sub-cortical channel comprises a series of four neurons: photoreceptor, bipolar cell, ganglion cell, and relay cell in the lateral geniculate nucleus. Cells are on-centre in one channel and off-centre in the other. Each cortical stage consists of a grid of neurons, and a square in the grid represents a single neuron. The sub-cortical stages converge onto each neuron in the first cortical stage, and all neurons in a given cortical stage converge onto each neuron in the next stage. **b.** In general, each neuron processes signals by weighting and summing the synaptic inputs, integrating the sum over time, and rectifying the resulting generator potential to produce an action potential rate. The exceptions are the photoreceptors, whose inputs are stimuli rather than synaptic inputs, and those neurons (photoreceptors and bipolar cells) whose output is a graded potential and which therefore do no rectification. **c.** The conversion between generator potential and impulse rate is taken directly from the work of Carandini and Ferster [24]. Generator potentials less than the action potential threshold produce zero impulse rate, and potentials greater than or equal to threshold produce an impulse rate proportional to generator potential. The circles indicate generator potential when stimulus contrast is zero. Neurons represented to the right of the origin have a spontaneous impulse rate, and the remainder (neurons in the first cortical stage) do not.

A block diagram of the model's structure is shown in Figure 1*a*. To be sensitive to motion, the model must have at least two spatially separated inputs. In the interests of simplicity, we start with exactly two inputs. These two inputs are assumed to be nearest-neighbour X-cell pathways. The anatomical correlate of the X-type retinal ganglion cell is the β cell, and Wässle et al. [19] have shown that nearest-neighbour β cells are almost always of differing sign – one is on-centre and the other off-centre. Accordingly, the sub-cortical portion of the model consists of two channels, one passing through an on-centre X-type ganglion cell and the other through the off-centre X cell that is its nearest neighbour. The stimulus to each channel is processed successively by a photoreceptor, bipolar cell, ganglion cell, and a relay cell in the dorsal lateral geniculate nucleus; each box in the sub-cortical channels represents one neuron. Input from the second eye is ignored.

The design of the model's cortical portion is heavily influenced by the finding that cortical cells can be separated into two types. Cross-correlation studies show that there are neurons in layer 4 and upper layer 6 that receive monosynaptic input from the geniculate [20]. Other cells receive their input from layer 4 cells [21], and are therefore separated by at least two synapses from the geniculate. We have therefore modelled primary visual cortex with multiple successive stages of processing. The number of cortical stages is set at three because, as we will show, the model takes at least this number of stages to produce responses like those in complex cells. The first stage represents layer 4 (and upper layer 6) of area 17. The model does not specify the nature of the later stages, but a strong possibility for the second stage is layers 2 and 3 [22], [23]. Neurons in a single stage are assumed to be aligned in a square grid; each member of the grid in Figure 1*a* therefore represents a single neuron. Each neuron receives convergent excitatory input from the neurons in the preceding stage, where the input is weighted with a Gaussian function centred on the recipient neuron. There are no connections within a stage, there is no feedback, and there are no inhibitory connections.

Part *b* of the figure shows signal processing within a single neuron. Synaptic inputs are summed and integrated over time to produce a generator potential; each neuron therefore acts as a low-pass temporal filter. For all cells other than photoreceptors and bipolar cells, this potential is rectified to obtain action potential rate. Figure 1*c* shows the function used to convert generator potential to action potential rate. The shape and gradient are taken directly from the work of Carandini and Ferster [24]. The dots on the function indicate the generator potential in the absence of a stimulus for three groups of cells. Sub-cortical stages are assumed to have a generator potential greater than threshold in order to account for their relatively high spontaneous impulse rates [25]. It will be shown below that neurons in cortical stage 1 are simple cells and neurons in later stages are more complex-like. Given that simple cells have little or no spontaneous impulse rate and that most complex cells have a non-zero rate [26], the stage 1 cells are assumed to be hyperpolarised relative to threshold and later stages to be moderately depolarised.

## Results

The most recognisable characteristic of a neuron in the visual system is probably its receptive field, the map of its response to small stimuli placed at a variety of visual field locations. We therefore start by showing receptive fields for the basic model. We then describe the other spatial characteristics of the model (orientation selectivity, spatial frequency selectivity) and spatiotemporal properties (direction selectivity). We finish by showing the emergence of complex cells.

### Receptive fields

**Figure 2:**
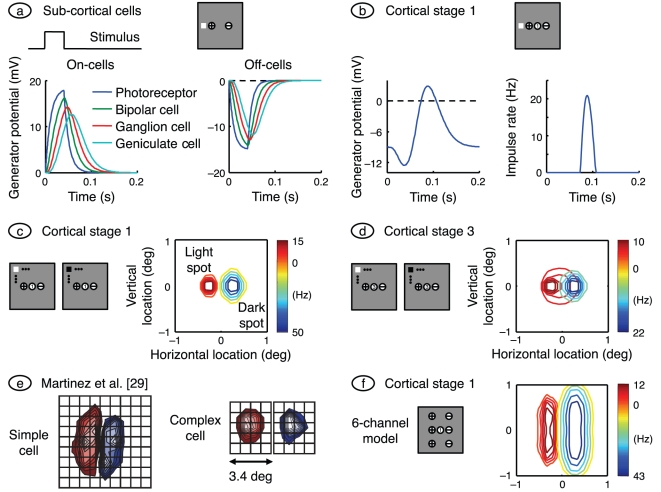
Responses to spots, and receptive field maps. **a.** The grey square represents the simulated (2°×2°) patch of visual field, and the *plus* and *minus* signs indicate the receptive field centres of the on- and off-centre channels, respectively (though not to scale). The white spot in the visual field represents a light square with a side length of 0.38° and with its centre 0.2° from the middle of the visual field patch, and the rectangular waveform at left indicates its time course. The graphs on the left and right show responses to this spot for on- and off-centre cells, respectively. All eight sub-cortical neurons are represented; only time-varying responses are shown, and the photoreceptor response on the left is inverted for easy comparison with the other traces. Time courses in the later sub-cortical stages are delayed relative to earlier stages because of the low-pass filtering action of all neurons. **b.** The resulting generator potential and impulse rate in the centrally located neuron of cortical stage 1 are shown on the left and right, respectively. **c.** This shows the receptive field of the model neuron centrally located in the first cortical stage. To produce it we followed the methods of Martinez et al. [29]. Light squares with a side of 0.38° and a duration of 40 ms were presented at the nodes of a 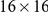 grid spanning the visual field patch, and impulse rate was calculated at 85 ms after stimulus onset. Red contours connect these responses, and blue contours connect the responses to dark spots. The colour bar at the right of the visual field gives the peak responses to the two spot polarities. **d.** The receptive field of the centrally located neuron in cortical stage 3 computed by the same method as for the stage 1 cell. **e.** Simple and complex cell receptive fields measured by Martinez et al., and reprinted by permission from Macmillan Publishers Ltd. The on- and off-subfields for the complex cell are spatially coincident (they are separated here for ease of comparison). **f.** Unlike the simple cell, the receptive field shown in part *c* shows little elongation. We rectified this fault by adding four more sub-cortical channels, as shown in the accompanying visual field map. Spot width here is 0.8°.

The receptive field of a stage 1 cortical cell is shown in Figure 2*c*, and the responses that contribute to it in parts *a* and *b* of the figure. The stimulus was brief (40 ms) as shown at the top of the figure. The grey square, also at the top of the figure, shows the 2°×2° patch of visual field modelled and the small light square within it represents the stimulus. The visual field patch also shows the middle of the receptive field of the on-centre (+) and off-centre (−) channels (though not to scale). The left side of part *a* shows the time course of neuronal responses in the on-channel; only the time-varying component of the response is shown and the time course of the photoreceptors, which hyperpolarise for light increments, is inverted for ease of comparison with the other responses. The peak response of each neuron is delayed relative to that of its predecessor, as expected of a cascade of low-pass filters. Responses of off-centre cells are shown on the right side of part *a*.

The on-responses are larger than the off-responses because the stimulus location is closer to the on-channel receptive field. As a consequence, the response of the cortical cell – chosen to be at the middle of the visual field patch – is dominated by the on-channel input, as shown in part *b* of the figure. The middle of the cortical cell's receptive field is shown in the visual field patch as a numbered circle; the number represents the cortical stage. The graph on the left shows cortical generator potential. The initial value of the potential, at the left side of the graph, is less than threshold (shown by the dashed line) as required by the *iceberg effect* in geniculo-recipient cells [27]. Impulse rate, shown on the right, is non-zero only when the generator potential rises above threshold.

Figure 2*c* shows the receptive field of the stage 1 cortical cell whose time courses are presented in part *b*. Maps such as this have been measured in the laboratory by presenting a spot stimulus at a succession of random locations and averaging the stimuli that precede impulses by a fixed delay [28]. We used a similar approach, with a delay – 85 ms – equal to the interval between stimulus onset and the peak of the response. Consistent with previous modelling work [8], [17], two subfields can be seen in the resulting receptive field. One is produced by light increments and is dominated by signals from the on-channel. The other is produced by light decrements and derives primarily from the off-channel. Compare this receptive field with that of the simple cell reproduced from Martinez et al. [29], on the left of Figure 2*e*. Like the simple cell, the model neuron has on- and off-subfields.

Figure 2*d* shows the receptive field of the neuron centrally located in cortical stage 3 of the model. There is considerably more overlap between the subfields here because the inputs from the previous cortical stages are purely excitatory (having been thresholded) and therefore disallow cancellation. One of the defining features of a complex cell is substantial overlap between on- and off-subfields. This is illustrated on the right of Figure 2*e*, where the subfields recorded by Martinez et al. are co-localised and have been separated for the purposes of illustration. It therefore appears that neurons in cortical stage 1 of the model represent simple cells and that neurons in stages 2 and 3 are more complex-like. Further evidence for this segregation is provided below.

There are two major deficiencies in the model receptive fields: they are spatially too confined and insufficiently elongated compared with those measured in the laboratory. The source of these faults is clear: a lack of sub-cortical inputs. Whereas real layer 4 neurons receive inputs from tens of geniculate relay cells [30], model neurons in the first cortical stage receive inputs from only two. We therefore added four sub-cortical channels to the basic model, as shown in the visual field map of Figure 2*f*. The resulting receptive field is substantially bigger and more elongated, as required.

### Orientation selectivity

**Figure 3:**
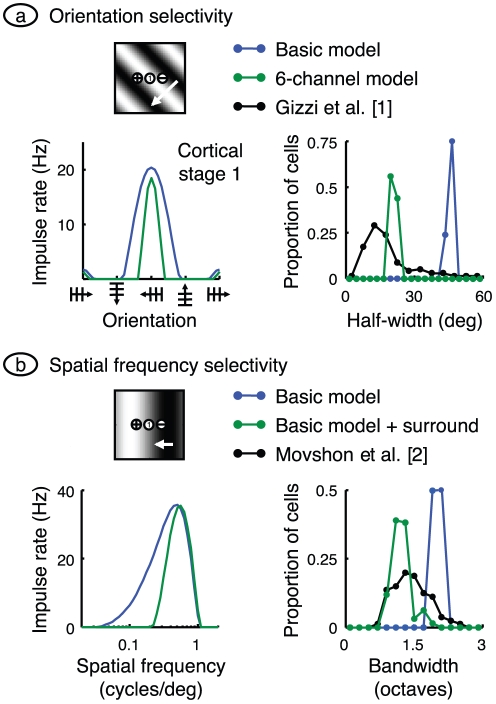
Spatial selectivity. **a.** Orientation selectivity in the model was tested by drifting a grating across the visual field with a variety of orientations. The methods were chosen to mimic those used by Gizzi et al. [1]: spatial frequency was optimal, 0.49 cycles/deg. Mean impulse rate for the cell centrally located in cortical stage 1 is shown at left in blue. The precision of orientation selectivity was found by measuring the half-width of this tuning curve at half-height. Half-width of all active stage 1 cells is shown by the blue frequency histogram at right, and compared with the data of Gizzi et al., shown in black. Clearly, the basic model has much poorer selectivity than that measured in the laboratory. This mismatch is partially remedied by using the six-channel model, as shown by the green curves. **b.** The basic model's spatial frequency selectivity was tested using the same methods as Movshon et al. [2], and is shown in blue. The tuning curve at left was computed by drifting a grating with optimal orientation, and a range of spatial frequencies. The response in the centrally located cell in cortical stage 1 was measured as the elevation of mean rate resulting from the stimulus. The half-width at half-height of all active stage 1 cells is shown as a frequency histogram at right, and compared with the data of Movshon et al., given by the black curve. The mismatch between the two histograms was largely removed by adding a surround mechanism to the sub-cortical channels, as shown by the green curves.

The model was designed to reproduce several fundamental properties of primary visual cortex, including sharp orientation selectivity. It can be seen from the receptive field shown in Figure 2*c* that the model must be at least coarsely orientation selective. The reasoning is as follows. Each bar of a grating aligned perpendicular to the subfields will produce inhibition from one cortical subfield that will reduce the excitation produced by the other subfield. The destructive interference will be less or absent when the grating is aligned with the subfields. Can the model quantitatively match the orientation tuning seen in the laboratory? The blue tuning curve on the left of Figure 3*a* was computed by drifting a grating across the receptive field of the cell at the middle of cortical stage 1 with a variety of orientations, and finding the mean impulse rate in each case. The neuron responds best to the orientation aligned with the subfields, and less well for other orientations.

The precision of orientation tuning is usually measured in the laboratory by finding the half-width of the tuning curve at half its height. The black symbols on the right of Figure 3a show the frequency histogram of half-width over a sample of simple cells [1]. Most neurons have a half-width less than 30°. To compare the model with these results we calculated the tuning curve for all active neurons in cortical stage 1 (see the Methods for our definition of *active*). Neurons in the basic model have half-widths clustered around 50°, a value substantially larger than for their empirical counterparts. The poor tuning in the model is due to the limitation to two sub-cortical channels. We therefore recalculated these data using the six-channel model. The results, shown in green, reveal a narrower tuning curve and reduced bandwidths on the left and right side, respectively, of Figure 3*a*.

### Spatial frequency selectivity

Another fundamental cortical property is selectivity for spatial frequency. Empirically, this property is examined by drifting an optimally oriented grating across the receptive field at a variety of spatial frequencies. The usual response measure is the fundamental Fourier amplitude of the impulse rate. The typical result [2], is that the neuron has an optimal spatial frequency, and that the response falls away rapidly on either side of the optimal value. Neurons in the first cortical stage of the basic model produce a similar result, as illustrated by the blue curve on the left side of Figure 3*b*. The existence of an optimal value is easily understood. When the light bar of the grating is over the on-subfield and the dark bar is simultaneously over the off-subfield the two phases of the grating contribute constructively in modulating the cell's impulse rate.

The tightness of tuning to spatial frequency can be assessed from the bandwidth of the tuning curve at half height. Data from one laboratory [2] are shown by the black symbols on the right of Figure 3*b*. They show the frequency histogram of tuning bandwidth for a sample of simple cells. Neurons are clustered around a bandwidth a little larger than 1 octave. The corresponding histogram for active neurons in the first cortical stage of the basic model is shown in blue. The model population is also tuned for spatial frequency but less so than for real neurons. This poorer tuning is due to the lack of a surround mechanism. In particular, very low spatial frequencies produce substantial surround signals that antagonise centre signals [31]. To apply the same effect here we added a surround mechanism to the basic model. The results, shown in green, produce a narrower tuning curve on the left side of Figure 3*b* and bandwidths close to empirical values, on the right.

### Direction selectivity: moving stimuli

**Figure 4:**
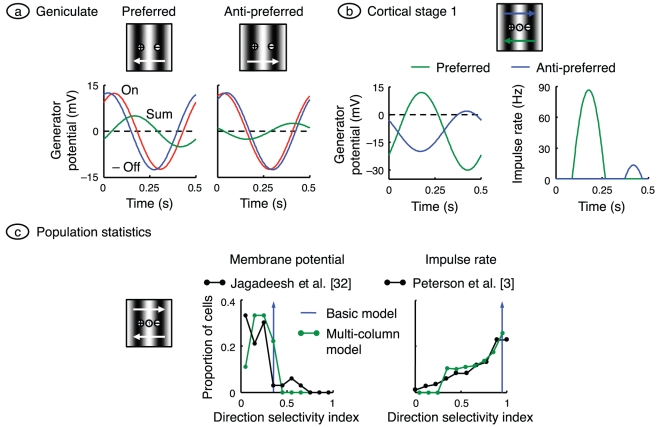
Direction selectivity: drifting gratings. **a.** Gratings with optimal orientation and spatial frequency were drifted across the visual field in both the preferred and anti-preferred directions. Geniculate generator potential in the on- and off-centre neurons is shown, along with their sum. Only time varying signals are shown and the off-centre signal is inverted, for ease of comparison. The on- and off-signals are closer in the anti-preferred case, resulting in a smaller sum. **b.** Generator potential and impulse rate are shown for the centrally located neuron in cortical stage 1 on the left and right, respectively. After thresholding, the anti-preferred response is much smaller than the preferred. **c.** Population responses in the model were compared with empirical responses by computing the direction selectivity index. Indices obtained from the generator potential are shown on the left, and are compared with the empirical data in Figure 9 of Jagadeesh et al. [32]. These authors calculated the index as 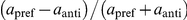 where *a_pref_* and *a_anti_* are the fundamental Fourier amplitudes for the preferred and anti-preferred directions, respectively; we follow suit. Indices obtained from impulse rate are shown on the right, and compared with those of Peterson et al. [3], who used 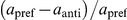. The basic model is represented by the blue vertical arrows: all neurons fall into the same histogram bin, to the right of the empirical data. To improve the match, the model was rerun with a range of delays between the on- and off-channels. The resulting histograms, shown in green, are closer to their empirical counterparts.

One of the key properties of neurons in primary visual cortex is direction selectivity: cells typically respond more strongly to a stimulus moved in one direction than in the opposite direction [5]. The direction of stimulus movement to which a cell responds best is called its preferred direction and the opposite direction will be referred to here as anti-preferred. Cortical cells in the basic model are direction selective, as shown in the right part of Figure 4*b*. Part *a* of the figure shows geniculate responses, to indicate how the selectivity arises. An essential requirement for direction selectivity is a temporal asymmetry between the two sub-cortical channels: the network must respond differently for motions in the two directions. This asymmetry is achieved in the model by assuming that one channel processes signals faster than does the other channel. In keeping with recent physiological evidence [16], the off-centre geniculate cells are assumed faster that their on-centre counterparts, with a latency difference of several milliseconds in the leading edge of the impulse response.

Part *a* of the figure shows the generator potential in both geniculate cells of the basic model for a single cycle of the grating stimulus. To better compare these two signals, the off-signal is inverted and only the time-varying signals are shown. The graph at left shows that when the grating is moving in the preferred direction the off-signal leads the on-signal. The graph at right, for stimulus motion in the anti-preferred direction, shows a much smaller phase difference between the two signals. Thus the sum of the on- and off-signals, which approximates the weighted sum formed by a cell in the first cortical stage, is smaller at right that at left. The reason for the directional difference is that for motion in the anti-preferred direction, the time taken for the stimulus to travel from the middle of the on-centre receptive field to the middle of the off-centre field is almost equal to the extra signal-processing time in the on-channel relative to the off-channel. Cancellation of the two inputs to the cortical cell is therefore almost complete, as shown by the sum curve.

Part *b* of the figure shows responses in the first-stage cortical cell whose receptive field lies midway between those of the sub-cortical channels. The generator potential in this cell is the result of weighting, summing, hyperpolarising, and low-pass filtering the curves in part *a*. The generator potential amplitude for stimulus motion in the preferred direction is greater than that for the anti-preferred direction, but not markedly so. The difference is much clearer after thresholding, to form the impulse rate on the right side of part *b*. Here the response for the preferred direction is much greater than for the anti-preferred direction. This is largely due to the iceberg effect, a phenomenon well documented from intracellular recordings of cortical cells [24].

Figure 4*b* shows the directionally selective response of a single cortical neuron in the model. To be more useful, however, the model should reproduce the observed diversity of directionality over a population of neurons. The extent to which a neuron is direction selective is typically defined by its direction selectivity index, a quantity with a value of zero when stimulus direction does not alter response, and one for the most direction-selective neurons. The direction selectivity index is calculated by drifting a grating across the receptive field and finding the spatial frequency and direction that maximise the fundamental Fourier component of the impulse rate. This preferred response is compared with the fundamental component when the same grating is drifted in the anti-preferred direction.

The frequency histogram of the direction selectivity index for cortical stage 1 in the basic model is shown in Figure 4*c*; the indices computed from membrane potential and impulse rate are shown on the left and right, respectively. All neurons in the basic model fall into a single bin, shown by the upward arrow. Compare this result with the histograms recorded by Jagadeesh et al. [32] on the left and Peterson et al. [3] on the right, and shown in black. Model cells are concentrated at the right end of the empirical data: the model is *too* direction selective. To make the direction selectivity more realistic we reduced the asymmetry between the two sub-cortical channels by decreasing their latency difference. The logic behind our procedure is as follows. The empirical data in Figure 4*c* come from many cells recorded across multiple cortical columns. It is to be expected that latency differences will vary from column to column. Correspondingly, we ran the basic model ten times, each time with a new value of the time constant difference between the two sub-cortical channels. In particular, we used differences uniformly distributed between 0 and 2 ms. The result is shown in green. The frequency histograms in this multi-column model more closely match the empirical data.

### Direction selectivity: stationary stimuli

**Figure 5:**
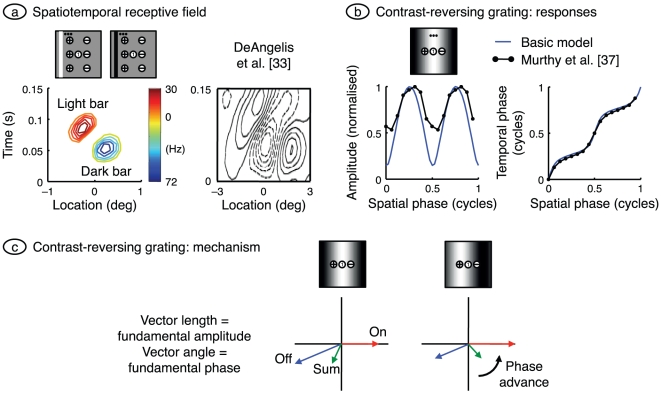
Direction selectivity: stationary stimuli. **a.** The spatiotemporal receptive field was calculated for the centrally located neuron in cortical stage 1 by presenting narrow bars of light and dark at a variety of locations, as illustrated in the visual field maps. Bars were 0.25° wide and were presented at 16 locations evenly distributed across the visual field patch. Bar duration was 40 ms. Contours connect responses to stimuli of the same polarity. The methods were chosen to match those used by DeAngelis et al. [33], whose results are shown at right (reprinted by permission from The American Physiological Society). The model produces slanted contours, as in the empirical data; the six-channel model was used because it yields elongated contours. **b.** The horizontal axis shows the spatial phase of a stationary grating whose contrast was varied sinusoidally in time; orientation and spatial frequency were optimal. The fundamental Fourier component in the resulting impulse rate was calculated, and its amplitude and temporal phase are shown on the left and right, respectively. Results from the basic model, shown in blue, are compared with those from the cell in Figure 4A and B of Murthy et al. [37]. Grating contrast in the model was set at 1 to obtain the best match in amplitude data. **c.** As shown in panel *b*, the model's response phase advances as the grating is shifted away from the off-centre input and towards the on-centre input. The vector diagram explains this finding. Vector length and angle give response amplitude and phase, respectively. Shifting the grating has opposite effects on the amplitude of the off- and on-centre inputs, advancing the phase of their sum. The sum represents the synaptic drive to the first-stage cortical cell at the middle of the receptive field patch, and the phase of this cell's impulse rate therefore advances as the grating shifts.

Direction selectivity has also been investigated using stationary stimuli. Figure 5*a* provides an example. The plot on the left shows the receptive field for which the horizontal axis gives the location of a stationary bar in the receptive field and the vertical axis gives time from the onset of the bar. The bar is optimally oriented and has a duration of 40 ms, matching the duration used by DeAngelis et al. [33]. The model's response can be understood by visualising a vertical line through the zero spatial location. The response to a dark bar (blue contours) appears earlier than that to a light bar (red contours) because of the faster processing in the off-channel. To the right of this line the map is dominated by responses to dark bars, because these preferentially stimulate the off-channel, and to the left by light bars, for which the on-channel dominates. The net effect is a set of contours slanted from lower left to upper right. The six-channel model is used here because it produces more elongated contours than does the basic model. The same slanting is seen in the empirical data on the right of Figure 5*a* [33], and is a signature of direction-selective neurons. The model therefore again reproduces the basic elements of laboratory observations.

Another consequence of the asymmetry of on- and off-channels should be noted from Figure 5*a*. The colour bar on the right side of the model plot shows the colour coding of impulse rate. It is clear from the colour bar that the response to dark bars is larger than that to light bars. This corresponds to the empirical finding that simple cells close to the central area are off-dominated [34]. Off-domination in the model occurs because the response in the off-centre channel is faster and therefore has a higher peak than that in the on-centre channel. The same effect is seen in the receptive fields plotted in Figure 2.

Stationary gratings have also been used to study direction selectivity. The idea here is that if the signal-processing is linear, the response to a moving stimulus should be predictable from the response to stationary stimuli placed at a series of locations across the receptive field [35], [36]. The filled circles in Figure 5*b* show the results of such an experiment [37]. A simple cell was stimulated with a stationary contrast-reversing grating. The horizontal axis gives the spatial phase of the grating and the left and right vertical axes give the amplitude and phase, respectively, of the response's fundamental Fourier component. The blue lines provide the same data for the basic model. The model was not adjusted to match the empirical data (apart from using a high grating contrast) and yet the model's temporal phase data match the laboratory data well. Also, like the simple cell, the model's amplitude data is always greater than zero and therefore shows no null.

The temporal phase on the right of Figure 5*b* advances with the grating's spatial phase. This is another signature of direction selectivity [35], [36] and a strong predictor of the direction to which a cell responds best: the preferred direction of a moving stimulus is that which “activates receptive-field positions with progressively shorter latencies” [37]. This is also true of the model. Increased spatial phase displaces a grating away from the off-centre input and towards the on-centre input, the preferred direction. This is a counter-intuitive finding in that this direction of displacement shifts the peak of the grating away from the low latency (off-) input and towards the high latency (on-) input. The mechanism underlying this result is shown in Figure 5*c*. Grating responses are shown on a vector diagram, where the length of a vector represents the (fundamental Fourier) amplitude in response to a contrast-reversing grating, and the direction of the vector represents temporal phase. The response of the on-input is arbitrarily pointed rightward and the off-response is almost 180° out of phase but has a slight phase advance representing its shorter processing time. The sum of these two vectors gives the synaptic drive of a cortical cell that weights these two inputs equally. When the grating is displaced in the preferred direction it activates the on-input more and the off-input less, producing a phase advance in both the sum vector and the cortical cell.

### Complex-like responses

Hubel and Wiesel [5] categorised neurons in primary visual cortex into simple and complex classes. One of the criteria for this categorisation was the form of the receptive field. Simple cells had subfields in which light increments evoked a response but decrements did not. These cells also had subfields in which a light decrement was required for a response, and on- and off-subfields were spatially separate. By contrast, a response could be obtained to both light on and off at each location in the complex cell receptive field. We have already shown in Figure 2 that model neurons at least partially replicate this behaviour. Cells in cortical stage 1 have clearly separated on- and off-subfields whereas cells in stage 2 (not shown) and stage 3 have partially overlapped subfields. Cells in the first cortical stage are therefore simple in character, corresponding to the finding that cortical cells connected monosynaptically to the geniculate are simple [38]. Cells in stages 2 and 3 are more complex-like.

**Figure 6:**
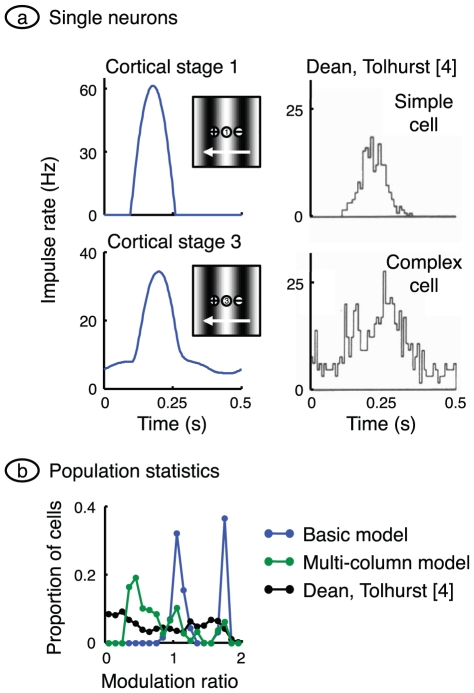
Complex-like responses. **a.** A grating of optimal orientation and spatial frequency, and a contrast of 0.25, was drifted across the receptive field patch. Impulse rate was computed for the centrally located neuron in cortical stages 1 and 3. Response measures were chosen to match those of Dean and Tolhurst [4] whose measurements from a simple cell and complex cell are shown at right (reprinted by permission from John Wiley and Sons Ltd.). The reduced impulse rate modulation in the stage 3 cell is due to rectification in previous stages, and static depolarisation. **b.** For each cell in their sample, Dean and Tolhurst calculated a modulation ratio equal to the Fourier fundamental amplitude of impulse rate divided by the mean rate. Their frequency histogram is shown in black. We have calculated the same ratio across all active cells in all three cortical stages of the basic model, and the resulting histogram is shown in blue. Stage 1 contributes the peak on the right and stages 2 and 3 together give the central peak. As in the laboratory, complex-like cells have a modulation rate close to or less than 1. A closer match between model and laboratory was obtained by allowing rectified geniculate impulse rates, as shown by the green histogram.

The use of drifting gratings provides another method for separating simple from complex cells [35]. Simple cells respond to a drifting grating with a modulated impulse rate: the rate rises and falls as each light bar crosses the receptive field. Complex cells respond with an increased impulse rate that is less modulated with time. Two examples from Dean and Tolhurst's work [4] are illustrated on the right of Figure 6*a*. Model neurons, illustrated at left, show similar behaviour. The stage 1 cortical cell fires only when the geniculate input exceeds threshold, and the cortical impulse rate is therefore strongly modulated in time. The third-stage cell has an unmodulated component in its impulse rate, for two reasons: it receives only rectified inputs from earlier stages, and the static polarisation in stages 2 and 3 is assumed to be depolarising.

These observations can be used to classify a neuron as simple or complex. Fourier analysis of the response to a drifting grating yields a fundamental component and a mean rate that quantify the modulated and unmodulated components, respectively. The modulation ratio is obtained by dividing the fundamental amplitude by the mean rate. Dean and Tolhurst [4] showed that simple and complex cells usually have a modulation ratio greater than and less than 1, respectively. The black curve in Figure 6*b* shows the frequency histogram of the modulation ratio compiled by these authors. The same graph also shows, in blue, the frequency histogram for all cortical stages in the basic model. There are two modes in the model's histogram, with the mode on the right due to cells in the first cortical stage. These cells have a modulation ratio greater than 1, reinforcing their classification as simple. Stage 2 and 3 are more complex-like in that their modulation ratios are close to or less than 1.

The model histogram has higher peaks than its empirical counterpart. Given that the basic model assumes no sub-cortical rectification of impulse rate, we investigated whether the inclusion of sub-cortical rectification could reduce this discrepancy. The published values described in the Methods indicate that geniculate cell centre mechanisms have a maximum contrast sensitivity of 450 Hz/contrast-unit. The model predicts that stimulation at the optimal spatial frequency, and surround antagonism, will lower this value to 280 Hz/contrast-unit. Given that X-type relay cells in the geniculate have a spontaneous impulse rate averaging 14 Hz [25], a grating contrast of greater than 0.05 will result in rectification. Laboratory studies almost always use a grating contrast greater than this value. We therefore assumed multiple columns, and that these columns differed – through variations in the spontaneous activity of their geniculate inputs – in the extent to which their inputs were rectified; spontaneous activity varied from 14 Hz up to a value which prevented rectification. Averaging modulation ratio across all columns produced the green curve, which is in better agreement with the empirical data.

## Discussion

Hubel and Wiesel [5] described two highly influential ideas, namely, that cortical orientation selectivity derives from aligned receptive fields in the lateral geniculate nucleus, and that complex cells receive their inputs from simple cells. The model described here goes beyond these ideas, and more recent modelling work, in several ways.

- Our model provides a mechanism for direction selectivity that is firmly based on empirical observations.
- We reproduce four fundamental properties – orientation selectivity, spatial frequency selectivity, direction selectivity, and the emergence of complex-like cells – in a single model.
- The model also shows that neurons sampled within and between columns possess these properties to varying extents, and that the model's population statistics largely match those measured in the laboratory.
- We show that dark stimuli tend to produce larger responses than do light stimuli, and that this off-domination follows naturally from the faster responses of off-centre geniculate inputs to cortex.

In what follows we discuss the geniculocortical synapse, direction selectivity, the role of inhibition in the model, the idea that simple cell responses are derivatives of their inputs, and sub-cortical connections.

### Geniculocortical synapse

The pivotal piece of circuitry in the model is the geniculocortical synapse. It is at this synapse that the model's orientation and direction selectivity both arise. Further, both of these properties depend on the convergence of on-centre and off-centre geniculate axons onto the same cortical cells. There are two important pieces of evidence supporting this assumption of convergence. First, Wässle et al. [19] showed that the anatomical substrate is available: the nearest neighbour of an X-type ganglion cell is nearly always of the opposite sign. Second, Reid and Alonso [38] showed that where an on-centre geniculate cell connects monosynaptically to a cortical cell, the geniculate centre mechanism and cortical on-subfield are almost invariably co-localised. Off-centre geniculate centre mechanisms and cortical off-subfields are similarly co-localised. The one piece of evidence remaining, then, is a direct demonstration that on- and off-centre geniculate cells project to the same cortical cell. In a tour de force of experimental technique, two laboratories [20], [39] have provided such evidence for simple cells.

### Direction selectivity

Early models for motion sensitivity assumed a quadrature relationship between the input sensors [11], [12]. For a 2 Hz stimulus, this requires that the output of one sensor be delayed by 125 ms relative to the other. Saul and Humphrey [13], who proposed that lagged and non-lagged geniculate cells could provide the sub-cortical substrate for direction selectivity, found that lagged cell latencies to grating stimuli averaged 70 ms longer than non-lagged cell latencies. By contrast, recent data shows that the on-centre and off-centre inputs to a cortical column differ in their arrival time by 3–6 ms [16]. We show here that the assumption of a latency difference of a few milliseconds is sufficient to generate strong direction selectivity in a simple feed-forward model. It seems, therefore, that future work on direction selectivity should consider much smaller latency differences than previously assumed.

### Inhibition

Inhibitory connections are not clearly evident in the model circuit, but inhibition plays a crucial role at two locations. The first is the sign-inverting synapse between photoreceptors and on-centre bipolar cells. It is this sign inversion that provides for the subsequent cancellation between on- and off-centre signals at cortical stage 1. The second role of inhibition is in hyperpolarising the cells in the same stage. It is this hyperpolarisation that sharpens selectivity through the iceberg effect. One piece of evidence for the assumed hyperpolarisation is that simple cells have little or no spontaneous activity [26]. Indeed, when a grating is used as stimulus, grating contrast has to be raised to a threshold level before any response is evoked from a simple cell [40]. Evidence that is more direct comes from intracellular recordings of simple cells, which show a hyperpolarised membrane potential in the absence of a stimulus [24]. It seems highly likely that this hyperpolarisation results from intracortical inhibition. Given the role of this inhibition in sharpening selectivity, it is not surprising that the blockade of inhibition results in a reduction of orientation selectivity [41].

There is a weakness in the model that we have not yet discussed. Orientation selectivity in real cortical neurons is largely contrast-invariant: increases in grating contrast do not markedly alter the tuning to orientation [42]. The same cannot be said of the model because increasing contrast will put more of the response above threshold and thereby broaden tuning. The addition of dynamic inhibition may remedy this fault. In particular, adding lateral inhibitory connections within each stage would introduce a hyperpolarisation that increases with stimulus contrast. This would help to preserve the iceberg effect.

### Cortex as differentiator

**Figure 7:**
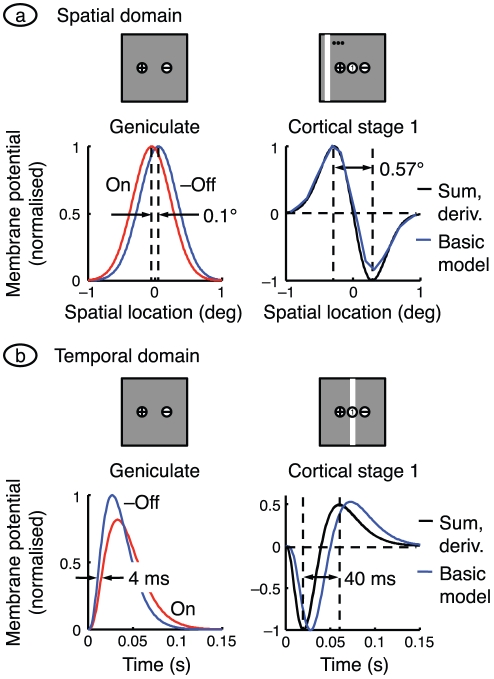
Approximating cortical responses with derivatives. **a.** The receptive field spatial profiles for the two sub-cortical channels in the basic model are shown on the left. The distance between peaks is set equal to the distance between neighbouring on- and off-centre X-type ganglion cells, and the off-centre signal is inverted. The graph on the right shows the sum of the two sub-cortical profiles and the spatial derivative of one of them (shifted so that the zero-crossing is centred). The sum and derivative are indistinguishable. The response of the centrally located neuron in cortical stage 1 of the basic model is also shown. It was calculated with the same bar stimulus used in Figure 5*a*, and the response is the generator potential 70 ms after bar onset. There is a good match between all three curves. **b.** The time courses on the left are impulse responses for the on- and off-centre geniculate cells in the basic model, with the off-centre curve inverted for ease of comparison. The sum of the on- and off-centre responses is shown in black on the right, along with the derivative of one of the responses (computed with the mean of the on- and off-centre time constants); the sum and derivative are indistinguishable. Also shown, in blue, is the time course of the membrane potential in the first-stage cortical cell at the middle of the receptive field patch. Its impulse response was calculated by delivering a very brief bar of light (width = 0.25°) at the middle of the patch. The black lines give the synaptic drive to the cortical cell and the blue line is relatively delayed because the cortical cell acts as a low-pass filter.

The assumption that on- and off-centre geniculate afferents converge onto the same cortical cell has a fascinating corollary, illustrated in Figure 7. The cortical cell adds the two opposite-signed inputs and is therefore effectively differencing similar spatial profiles. It is estimated in the Methods that the distance between neighbouring on- and off-centre receptive fields is 0.1° at the eccentricity of interest (11°), as shown on the left of part *a* of the figure. This is substantially less than the centre mechanism radius of a geniculate afferent, 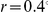. Accordingly, the cortical receptive field spatial profile is the difference between similar spatial profiles separated by a relatively small distance, and is therefore approximately proportional to the spatial derivative of a single geniculate centre mechanism. The black curves on the right show the sum of the on- and off-inputs, and the (centred) derivative of one of them. The two curves overlie each other. The blue curve, showing the membrane potential of the first-stage cortical cell briefly stimulated with light bars (as in Figure 5*a*), matches well with the black curves. The centre mechanism is assumed to have a Gaussian profile; computing its derivative shows that the cortical cell's on- and off-subfields are separated by 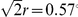, as shown. This calculation helps to explain why the subfield separation in the cortex is substantially larger than the spacing of neighbouring retinal cells.

This idea extends to the temporal domain. The impulse responses of the on-centre and off-centre geniculate cells are plotted at the left of Figure 7*b*. These responses are gamma densities with shape factor 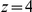 because they result from a cascade of four first-order low-pass filters. The on- and off-centre functions have time constants of 11 and 9 ms, respectively, and because of the closeness of these values the difference between them can be approximated by differentiating one of them with respect to time constant. The right side shows, in black, the sum of the two geniculate inputs and the derivative of a gamma density with a time constant midway between that of the two inputs, 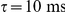. The distance between the trough and peak (calculated by differentiating with respect to time) is 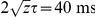.

Also shown, in blue, is the membrane potential of the first-stage cortical cell at the middle of the visual field patch. This approximation to the basic model's impulse response was generated by presenting an optimally oriented bar very briefly at the middle of the patch. This response is similar in shape to the derivative function but is slower because it gives the cell's output rather that its synaptic drive, and therefore includes extra low-pass temporal filtering. These results together explain a counter-intuitive result. Whereas the off-input to cortex precedes the on-input by only a few milliseconds, the off-peak in the spatiotemporal receptive field (Figure 5*a*) leads the on-peak by tens of milliseconds.

### Sub-cortical connections

According to the calculations in the Methods, there are over 200 X-type ganglion cells in the 2°×2° visual field patch used here. The connection of just two (or six) of those cells to the first cortical stage of the model is therefore highly selective, a selection that will enhance orientation selectivity in cortex. Alonso et al. [30] have shown that layer 4 cells connect to only about one third of the geniculate relay cells available to them. Given the narrow orientation of these cells, it is natural to assume that the choice of connection is that which enhances orientation selectivity. How is the choice made? One possibility is the following. During the developmental period, the first two relay cells making a connection are likely to be driven by nearest neighbours in the retina, which almost certainly have centres of opposite sign. These two connections will establish broad orientation tuning. Other retinal neighbours will then attempt to contact the cortical cell via a relay cell. If Hebbian principles operate they will only succeed if their own firing enhances impulse rate in the cortical target. Only connections that enhance the existing orientation tuning will survive.

The receptive field of a cortical stage 3 neuron, shown in Figure 2*d*, indicates that the on- and off-subfields only partially overlap. This fails to match the complete overlap in the complex cell subfields shown in part *e* of the figure. The reason for the incomplete overlap in the model is clear: on-centre sub-cortical inputs are segregated from off-centre inputs, regardless of the number of channels. It has recently been shown, by contrast, that on-centre inputs to a given cortical column are dispersed among the off-centre inputs [15]. It would be of considerable interest to discover whether the Hebbian process described above can produce intermixing of on- and off-inputs in the model, and complete overlap of stage 3 subfields.

## Methods

### Model equations

Each neuron in the model is represented by a single nonlinear differential equation, and time courses in the model are obtained by simultaneous numerical integration of the equations for all neurons. We here derive the equations for the general model, that is, the model that includes multiple sub-cortical channels, surround mechanisms, and sub-cortical rectification. The pivotal variable in the model is the membrane potential at the initial segment of a neuron's axon. This is the potential that generates action potentials and is therefore referred to here as the generator potential, *p*. The growth rate of the generator potential depends on the postsynaptic potentials, *v_k_*, from multiple synapses driving the neuron. The contribution of each postsynaptic potential is weighted by a gain *g_k_* that declines with the distance between the receptive fields of the neuron and its presynaptic driver. The generator potential growth rate also depends on a static polarisation, 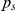, that is independent of the visual stimulus. The static polarisation is responsible, for example, for the high spontaneous impulse rate in sub-cortical neurons and for the hyperpolarisation that produces the iceberg effect [27] in cortical simple cells. Quantitatively, the time derivative of the generator potential is
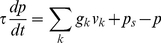
(1)The last term in this equation ensures that generator potential grows with a time constant *τ*; the equation therefore guarantees that the neuron acts as a low-pass filter. The conversion from generator potential, 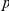, to action potential rate, *a*, is shown in Figure 1*c* and is taken from the work of Carandini and Ferster [24]. It takes the form of a rectifier,
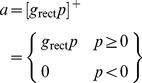
(2)where *p* is defined to be the difference between membrane potential and action potential threshold, and *g*_rect_ is the gain of the generator function.

Assume that Equation 1 applies to a neuron in stage *z* of the model. We need to relate it to generator potentials in the previous stage, 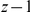, if time courses are to be computed. We make the simplest assumption: postsynaptic potential is proportional to impulse rate in the presynaptic neuron, and the conversion function is the inverse of that in Equation 2 (any difference in the proportionality constants at the initial segment and the synapse can be absorbed into the gain *g_k_*). The conversion from generator potential in a neuron at stage 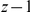 to postsynaptic potential in the target neuron at stage *z* is then given by
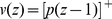
(3)Equation 1 then becomes
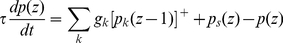
(4)We generalise this equation by including the dependence on time, *_t_*, and visual field location, 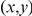, and by showing the sum term as a spatial convolution:
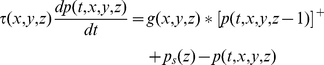
(5)where
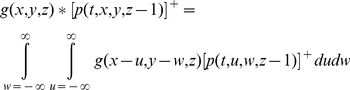
(6)This equation needs three modifications for the sub-cortical stages of the model. First, the driver at the first stage is the visual stimulus, 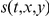, not a presynaptic neuron. Second, the sign 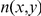, of the driver depends on whether the neuron being modelled is on-centre or off-centre. Photoreceptors hyperpolarise to light, and the first synapse for the on-centre channel is sign inverting. For computational simplicity we assume that the photoreceptors driving on-centre channels depolarise to light. The sign of the first term on the right of Equation 5 is then positive for on-centre and negative for off-centre channels. Third, the neurons presynaptic to bipolar and ganglion cells do not produce action potentials, so there is no rectification. Thus:
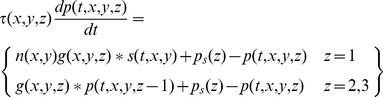
(7)The gain function, *g*, takes several forms depending on the stage. For the sub-cortical stages it includes centre-surround antagonism, implemented as a difference of Gaussians. For computational simplicity, all sub-cortical spatial convergence is collapsed into the first stage:
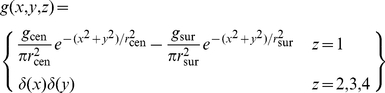
(8)where *r* stands for radius, and *δ* is the Dirac delta function. For cortical stages, the gain function is purely Gaussian:
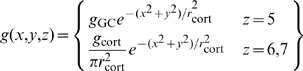
(9)where *GC* stands for geniculocortical. Substitution of Equation 8 into Equations 5 and 7 results in the following simplification:
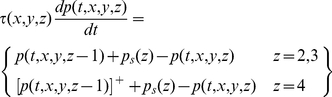
(10)The sub-cortical pathways can be divided into channels. Assume that there are *m* channels. The *i*th channel (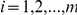) is defined by the location of the middle of its receptive field, 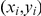, its sign, 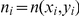, and its time constant:
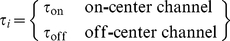
(11)It is convenient, then, to recast the equations for the sub-cortical stages using subscripts rather than function arguments:
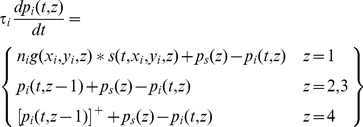
(12)The input to the cortex is then spatially discrete:
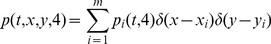
(13)A further simplification can be achieved by considering sub-cortical resting potentials. We assume that these potentials are above threshold, in order to produce the spontaneous impulse rate observed in ganglion and geniculate cells [25], and that the resting potentials are the same for all stages. Resting potential is calculated by setting the stimulus and time derivatives to zero. Solution of Equation 12 then yields
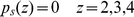
(14)Denoting the cortical time constant as *τ*_cort_, the model's defining equations can then be stated in their final form:
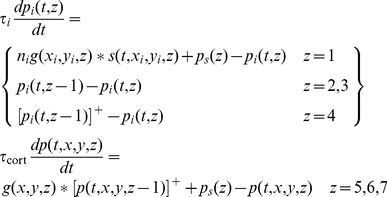
(15)


### Resting activity

Resting activity in a neuron is important because it can determine whether signals passing through the neuron are rectified. Spontaneous impulse rate in the model is determined, in part, by the static polarisation, 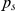. We assign the following values to this parameter:
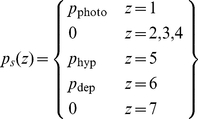
(16)Resting activity can be determined by setting both the stimulus and the derivatives to zero. From Equation 15, resting potential in the first five stages is
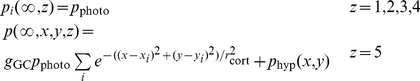
(17)The static hyperpolarisation 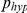 in stage 5 is set sufficiently negative that the resting potential is also negative. This ensures that cells in that stage, the first cortical stage, have no spontaneous impulse rate, in keeping with most simple cells [26]. Thus, from Equation 15,
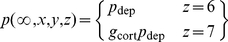
(18)From Equation 2, the spontaneous impulse rate in those cells that produce action potentials is
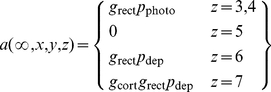
(19)


### Parameter settings

There follows a description of the model parameters and how they were set. Given the variability of the measurements, only two significant places are retained. Table 1 provides a glossary of the parameters and their values.

**Table 1:** Glossary of symbols.

Symbol	Parameter	Value	Unit
*_c_*	Contrast	Variable	None
*g*_cen_	Centre mechanism contrast sensitivity	62	mV contrast-unit^−1^
*g*_cort_	Intracortical gain	1	None
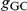	Geniculocortical gain	4.21 (2 channels); 1.47 (6 channels)	None
*g*_sur_	Surround mechanism contrast sensitivity	48	mV contrast-unit^−1^
*g*_rect_	Gain of generator function	7.2	Hz/mV
*i*	Index of sub-cortical channel	1, 2, …, *m*	None
*m*	Number of sub-cortical channels	Variable	None
*n_i_*	Sign of *i*th sub-cortical channel	1 (on-channel); −1 (off-channel)	None
*ω_s_*	Stimulus spatial frequency	Variable	radians/deg
*ω_t_*	Stimulus temporal frequency	2π×2	radians/s
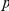	Generator potential	Variable	mV
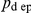	Static polarisation, cortical stages 2, 3	0.646	mV
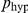	Static polarisation, cortical stage 1	−25.5 (*x* = *y* = 0)	mV
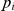	Generator potential in *i*th sub-cortical channel	Variable	mV
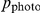	Sub-cortical static polarisation	1.94	mV
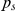	Static polarisation	Variable	mV
*ϕ*	Spatial phase	Variable	radians
*r*_cen_	Radius of centre mechanism	0.4	deg
*r*_cort_	Radius of cortical convergence	2.8	deg
*r*_sur_	Radius of surround mechanism	1.1	deg
*t*	Time	Variable	s
*τ*_cort_	Time constant of cortical cells	10	ms
*τ_i_*	Time constant in *i*th sub-cortical channel	*τ*_on_ (on-channel); *τ*_off_ (off-channel)	ms
*τ*_off_	Time constant of off-centre cells	9	ms
*τ*_on_	Time constant of on-centre cells	11	ms
*θ*	Stimulus orientation	Variable	radians
*x*	Horizontal position in visual field	Variable	deg
*x_i_*	Horizontal position of channel *i*	Variable	deg
*y*	Vertical position in visual field	Variable	deg
*y_i_*	Vertical position of channel *i*	Variable	deg
*z*	Index of processing stage	1, 2, …, 7	None

The table provides a glossary of symbols used in this paper. Values are given to three significant places.

### Spatial parameters

#### Location and size of visual field patch

For reasons explained below, we assume a visual field patch centred on the horizontal meridian, and 11° from the central area. The size, 2°×2°, is intended to span a substantial fraction of a typical cortical receptive field.

#### Retinal magnification factor

We use the value calculated by Hughes [43], 0.20 mm/deg. The retinal patch therefore had an eccentricity of 11×0.20 = 2.2 mm.

#### Concentration of X-type ganglion cells

We use the data of Stein et al. [44] whose method resulted in no detectable concentration difference between wet and dry retinal samples. At 2.2 mm eccentricity the mean of nasal and temporal β cell concentrations was 1275 cells/mm^2^. Given that β cells are the morphological correlates of X-type ganglion cells, the concentration of X cells is then 1275×(0.20)^2^ = 51 cells/deg^2^. Stein et al. made their measurements along the nasotemporal axis, and we have correspondingly placed our retinal patch on the horizontal meridian.

#### Distance between X-type ganglion cells

Wässle et al. [19] examined the packing of same-sign β cells. They found a continuum between square and hexagonal arrays. We make the simpler assumption, a square array, and also assume that there are equal numbers of on- and off-centre X cells. The spacing of same-sign X cells is then 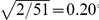.

#### Distance between opposite-sign X-type ganglion cells

The most relevant data come from Wässle et al. [19], who measured nearest-neighbour distances for both on- and off-centre β cells. The mean distance between nearest neighbours was 43 µm, and for nearest neighbours of the same sign the distance was 88 µm, with a ratio of 0.49. We assume that nearest neighbours are of opposite sign and that the same ratio holds at the eccentricity of interest here. Multiplying the ratio by the mean spacing of same-sign cells, obtained above, yields a distance between opposite-sign X cells of 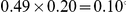.

#### Size of X-type lateral geniculate cells

These are taken from the work of Saul and Humphrey [13]. The mean eccentricity for their sample was 11°, which is the reason for choosing this eccentricity for the visual field patch. From their mean radii of the centre and surround mechanisms of (non-lagged) X cells, 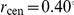 and 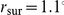.

#### Cortical magnification factor

This factor, 0.45 mm^2^/deg^2^ is taken from the measurements of Tusa et al. [45] at 11° eccentricity along the horizontal meridian.

#### Cortical density of neurons

Beaulieu and Colonnier [46] found 78,440 neurons under each mm^2^ of binocular cortex. To obtain the linear cell density in the model we apply the following operations. First, this value is multiplied by the cortical magnification factor to convert it to degrees. Second, the model contains only excitatory neurons; assuming that all other neurons contain GABA, we multiply by 0.794 to eliminate them [47]. Third, we divide by 3 to obtain the density per stage. Finally, we assume that neurons are arranged in a square array and therefore take the square root to find the linear density. The result is 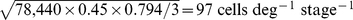.

#### Radius of cortical cell receptive field

Gardner et al. [48] measured subfield length in a sample of simple cells. The geometric mean of their sample was 5.5°. Halving this value gives a radius of 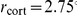.

#### Distance between same-sign X-type ganglion cells

The general model assumes more than one ganglion cell of the same centre sign. The distance between ganglion cells of the same sign can be as small as 0.2°, as described above, but can also be much larger, as indicated by the radius of the cortical cell receptive field. We chose a compromise distance of 0.75°: this produces an elongated subfield that largely fits into the 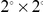 receptive field patch.

### Temporal parameters

#### Cortical time constant

There is a problem in estimating the time constant for model cells: the neurons modelled are inhomogeneous in their temporal properties. Phototransduction, for example, includes the time required for a series of reactions not present in following cells. We have therefore taken a pragmatic approach. The model assumes that temporal processes can be lumped into one first-order low-pass filter for each neuron. The impulse response of a series of *z* low-pass filters with time constant *τ* peaks at 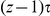. For a first-stage cortical cell (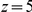), this peak time is 4*τ*. Given that simple cell impulse responses peak at values as low as 40 ms [49], 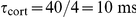.

#### Sub-cortical time constants

It has recently been shown that off-centre X-type geniculate cells lead their on-centre neighbours. In particular, the leading edge of the impulse response in off-cells precedes that in on-cells by a mean of 3 ms when measured at 40% of maximum response [16]. We set time constants in the two sub-cortical channels as follows: 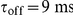, 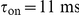. Figure 7*b* shows that the model approximates the empirical finding.

### Intensive parameters

#### Generator gain

The form of the generator function (Figure 1*c*) and its gradient, 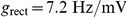, are taken directly from the work of Carandini and Ferster [24].

#### Geniculate contrast sensitivity

This parameter can be calculated by integrating the centre mechanism's spatial profile over both dimensions:
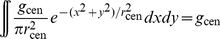
(20)We set this equal to the contrast sensitivity of the X-type ganglion cell centre mechanism, 620 Hz/contrast-unit (from the 2 Hz data in Figure 12 of Frishman et al. [50]), multiplied by the attenuation between retina and geniculate, 0.73 (from Figure 5A of Kaplan et al. [25]). Finally, converting from Hz to mV, *g*_cen_ is given by:
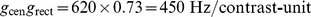
(21)


#### Surround contrast sensitivity

We use Saul and Humphrey's [13] measurements of mechanism strength, 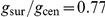.

#### Cortical contrast sensitivity

The contrast sensitivity of stage 1 cortical cells is best determined from the responses of simple cells to gratings of optimal orientation and spatial frequency. We used the membrane potential measurements of Carandini and Ferster [24] because they avoid the complications of action potential threshold. Dividing response amplitude by contrast, the maximum gradient for the three simple cells in their Figure 13 averages 70 mV/contrast-unit. The geniculocortical gain, 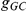, was set so that the contrast sensitivity of stage 1 cortical cells replicated this value.

#### Static hyperpolarisation

This parameter was estimated from the work of Anderson et al. [51]. From their Table 1, the median difference between threshold and resting potential in nine simple cells is 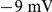. The second equality in Equation 17 was solved for 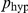 by setting the left side to this value.

#### Intracortical gain

There is little evidence for consistent contrast sensitivity differences between simple and complex cells [40]. We therefore assumed unity gain between one cortical stage and the next. The parameter *g*_cort_ is then given by:
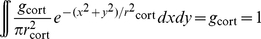
(22)


#### Static depolarisation

Cells in primary visual cortex have a mean spontaneous impulse rate of 3.1 Hz [26]. From Equation 19, the mean impulse rate in model cortical cells is 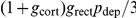. This value was set to 3.1 Hz and solved for 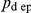.

### Stimuli

There is no surround antagonism in the basic model. By way of compensation, stimuli are defined in terms of local contrast rather than luminance. Local contrast is obtained by finding the difference between local and background luminance, and dividing the difference by background luminance. We use three types of stimuli: gratings, spots, and bars.

#### Grating

The equation for a drifting grating is
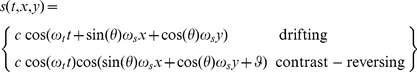
(23)where
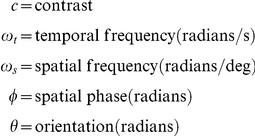



#### Spot, bar

For these stimuli
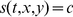
(24)during stimulus presentation and in the visual field area covered by the stimulus. Otherwise 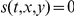.

Stimulus parameters matched published values as far as possible. Neurons in primary visual cortex are typically broadly tuned for temporal frequency, and 2 Hz is often used in published work; we also use this value. We show empirical data in several of our figures. Each of the quoted studies used a range of grating contrast, typically 0.25–0.5. Unless otherwise stated, we use a grating contrast of 0.3. Spot and bar contrasts are usually not stated in the literature; we use 1 for light stimuli and 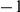
for dark.

### Computation

All simulations were performed in Matlab (The MathWorks, Inc); the model equations were numerically integrated using Matlab's *ode45* function. We reduced the risk of coding errors in two ways. First, the two authors implemented the model equations independently before comparing results. Second, for low stimulus contrasts the model's equations are linear up to the production of impulses in cortical stage 1 neurons. We solved these equations analytically and ensured that the numerical and analytical solutions agreed.

When compiling population statistics we needed some way of deciding which neurons should be excluded because of insufficient activation by the stimulus. This process of exclusion has a correlate in the laboratory: the experimenter encounters a new cell with the electrode and decides not to study it if it is insufficiently active. Our criterion was as follows. A grating with optimal orientation and spatial frequency was drifted across the receptive field at 2 Hz. A neuron was excluded from analysis if the resulting elevation of its mean impulse rate was less than a criterion level. Following Romo et al. [52], we set the criterion at 5 Hz.
